# Quality of life in sexagenarians after aortic biological vs mechanical valve replacement: a single-center study in China

**DOI:** 10.1186/s13019-020-01143-w

**Published:** 2020-05-12

**Authors:** Li-Wen Wang, Ning Xu, Shu-Ting Huang, Liang-Wan Chen, Hua Cao, Qiang Chen

**Affiliations:** 1 Department of Cardiac Surgery, Fujian Maternity and Child Health Hospital, Affiliated Hospital of Fujian Medical University, Fuzhou, China; 2 Department of Cardiovascular Surgery, Union Hospital, Fujian Medical University, Fuzhou, China

**Keywords:** sexagenarian, Prosthetic valve replacement, Quality of life, Anxiety

## Abstract

**Objective:**

This article aimed to study the quality of life and anxiety of sexagenarian patients who underwent aortic biological vs mechanical valve replacement in a single center in China.

**Methods:**

The clinical data of 78 patients aged 60 to 70 years who underwent aortic prosthetic valve replacement were retrospectively analyzed in our hospital from June 2017 to February 2018. Patients were divided into two groups depending on the type of prosthetic valve they received (biological valve group vs mechanical valve group). The SF-36 was completed by all patients at discharge and at one-year follow-up, and the cardiac anxiety questionnaire (CAQ) was also completed at one-year follow-up.

**Results:**

There was no statistically significant difference between the two groups in general clinical data or SF-36 score at discharge. However, at one-year follow-up, the SF-36 scores were significantly higher in the biological valve group than in the mechanical valve group, and the CAQ scores in fear and anxiety, avoidance and attention in the mechanical valve group were significantly higher than those in the biological valve group.

**Conclusions:**

Based on the postoperative quality of life and anxiety scores of sexagenarian patients who underwent biological vs mechanical valve replacement in this study, a biological valve has more value than a mechanical valve for sexagenarians undergoing aortic valve replacement.

## Introduction

Aortic valvular disease is one of the most common acquired heart diseases, and prosthetic valve replacement is the main treatment. The types of prosthetic valves available clinically are divided into prosthetic mechanical and biological valves [1, 2]. The advantages of biological valves are that they do not require anticoagulation therapy over the lifetime of the patient after surgery, and patients are not affected by the noise of the valves. However, biological valves still have a relatively short service life, and some patients may face the risk of secondary surgery [3]. At present, it is a relatively common opinion that mechanical valves be chosen for young patients while biological valves are chosen for elderly patients in Chinese clinical practice. However, there is no consensus on the type of valve that should be used in patients aged 60–70 [4, 5]. The choice of artificial valve is related to many factors, such as patient age, sex, education, financial ability, general physical condition, medical compliance, and willingness to accept anticoagulation therapy. This study attempted to use quality of life as a selection factor by comparing and analyzing the quality of life of sexagenarian patients who underwent aortic biological or mechanical valve replacement, providing a reference for the selection of valve types in this specific age group.

## Materials and methods

### General information

This study retrospectively analyzed the clinical data of 78 patients admitted to our hospital who underwent aortic prosthetic valve replacement from June 2017 to February 2018. All the patients were divided into two groups according to the prosthetic valve type that the patient chose: the mechanical valve group (40 patients) and the biological valve group (38 patients). Prior to the valve replacement, the patient and his/her family were informed in detail of the special properties of the two kinds of prosthesis valves, their characteristics of use, the need for post-operative anticoagulation therapy, and the durability of the prosthesis valve. After full communication with the doctor, the patient and his/her family selected the corresponding prosthesis valve according to their own situation and their own will. The general information of all patients is shown in Table 1. The inclusion criteria were as follows: patients aged 60 to 70 years old, patients undergoing conventional median thoracotomy, and patients needing conventional aortic biological or mechanical valve replacement. The exclusion criteria were as follows: patients of other age groups, patients undergoing other heart surgery or simultaneous coronary artery bypass grafting, patients with cardiac function III-IV, patients with other important organ dysfunction, patients who died of unexplained causes within 1 year after surgery, and patients who were not willing to participate in this study.

**Table 1:** Comparison of general data between the two groups

	Biological AVR	MechanicalAVR	*P* values
cases	40	38	
age	55.6 ± 4.3	54.8 ± 5.1	0.876
gender	24/16	21/17	0.672
Cardiac function			
I	0	0	0.880
II	28	26	
III	12	12	
IV	0	0	
The lesion type			
stenosis	13	15	0.795
insufficiency	9	7	
stenosis with insufficiency	18	16	
Ejection fraction	57.9 ± 11.8	55.3 ± 13.6	0.682
The living environment			
rural	29	27	0.887
city	11	11	
marriage			
married	37	35	0.54
unmarried	1	0	
Death of a spouse	2	3	

### Questionnaires

The Medical Outcomes Study Short Form 36 (SF-36) health scale is characterized by good reliability and validity and is widely used to evaluate the quality of life of all kinds of people [6]. It consists of 36 items, and the eight aspects of health-related quality of life are divided into physical and mental health: 1. PF: physical functioning; 2. RP: role physical; 3. BP: bodily pain; 4. GH: general health; 5. VT: vitality; 6. SF: social functioning; 7. RE: role emotional; and 8. MH: mental health [7, 8]. Each field is explored by different questions, and each question is answered with a different frequency level and a corresponding score. Respondents must choose one of them as the answer, and the final score is calculated based on these answers.

The cardiac anxiety questionnaire (CAQ) is a scale developed by the Department of Psychology of West Virginia University to assess the severity of cardiovascular anxiety symptoms [9]. It consists of 17 items with 3 subscales, including fear (e.g., chest pain and other related sensations), avoidance (e.g., activities believed to cause cardiac symptoms) and attention (awareness of heart-focused sensations such as the heartbeat). Each dimension contains five to eight items, for a total of 17 items. According to the frequency of symptoms, there are 5 levels of responses: 0 for “never”, 1 for “rarely”, 2 for “sometimes”, 3 for “often”, and 4 for “always”. The higher the score is, the more serious the related psychological disorder [10].

All participants were asked to complete the questionnaire independently, and researchers were allowed to ask questions but were not allowed to interfere with the participants’ answers. The SF-36 was completed at discharge and at one-year follow-up, and the CAQ was completed at one-year follow-up.

### Statistical analysis

SPSS 18.0 was used for statistical analysis. According to the anxiety condition after discharge of the two groups of patients in the preliminary survey, we defined α = 0.05. On the bilateral inspection, the study efficiency was 90%, and the sample sizes of the two groups were calculated as 40 and 38, respectively. The quantitative data are expressed as the mean ± standard deviation, and the continuous data were tested for normal distribution. An independent sample t test was used for those data conforming to a normal distribution, while the Wilcoxon test was used for those data not conforming to a normal distribution. Quantitative data were compared between groups by the chi-square test. *P* < 0.05 was considered statistically significant.

## Results

As shown in Table 1, there were no significant differences between the two groups in age, sex, cardiac function, living condition or marital status. There was no significant difference in the SF-36 score between the two groups at discharge (Table 2). These results indicated that the two groups of patients were homogeneous and had intergroup comparability.

**Table 2:** Preoperative comparison of SF-36 scores

	Biological AVR	Mechanical AVR	P values
Physiological function	63.5 ± 12.1	64.3 ± 11.3	0.647
Role physical	68.3 ± 11.8	68.4 ± 15.2	0.728
Bodily pain	86.2 ± 13.7	84.6 ± 10.6	0.682
General health	60.3 ± 14.8	61.2 ± 13.8	0.572
Vitality	63.1 ± 17.2	62.5 ± 14.1	0.389
Social functioning	65.4 ± 13.2	67.3 ± 16.6	0.829
Role emotional	67.8 ± 13.6	66.5 ± 17.8	0.563
Mental health	61.6 ± 14.2	60.4 ± 12.6	0.601

When comparing the SF-36 scores at one-year follow-up, the results showed that the scores of physical functioning, role physical, general health, vitality, social functioning, role emotional and mental health in the biological valve group were significantly higher than those in the mechanical valve group, which indicated that the quality of life in the sexagenarians in the biological valve group was higher than that in the mechanical valve group (Table 3). The comparison of CAQ scores at one-year follow-up showed that the fear and anxiety, avoidance and attention scores of the patients in the mechanical valve group were significantly higher than those in the biological valve group, which indicated that the patients in the mechanical valve group showed more anxiety than those in the biological valve group at one year after surgery (Table 4).

**Table 3:** Comparison of SF-36 scores one year after surgery

	Biological AVR	Mechanical AVR	P values
Physiological function	83.4 ± 15.4	70.3 ± 11.3	0.035
Role physical	86.5 ± 18.7	76.5 ± 13.6	0.024
Bodily pain	67.2 ± 10.4	66.8 ± 12.4	0.712
General health	84.2 ± 15.2	73.6 ± 15.5	0.032
Vitality	83.6 ± 16.7	72.7 ± 14.2	0.021
Social functioning	86.5 ± 15.5	75.2 ± 17.3	0.040
Role emotional	88.2 ± 18.4	76.6 ± 15.9	0.018
Mental health	83.7 ± 16.2	71.6 ± 14.5	0.029

**Table 4:** Comparison of CAQ scores at 1 year after surgery

	Biological AVR	Mechanical AVR	P values
Fear and anxiety	7.7 ± 3.6	14.8 ± 5.5	0.027
Avoidance	5.2 ± 2.8	9.1 ± 3.2	0.039
Attention	3.3 ± 2.2	9.6 ± 4.1	0.018

## Discussion

Aortic valve disease is one of the most common heart valve diseases and has been on the rise in recent years. Moderate to severe aortic valve disease is often associated with several symptoms, including chest pain, syncope, heart failure, and even sudden death, and requires surgical intervention [11]. At present, the most common surgical method is prosthetic valve replacement, and the types of artificial prosthetic valves commonly used in the clinic can be divided into biological and mechanical valves [12]. The advantages of biological prosthetic valves are that they do not require anticoagulation therapy or monitoring of the coagulation status for the lifetime of the patient, and patients are not affected by the noise of the valve. However, the biological prosthetic valve has the disadvantage of a relatively short service life, which also limits its application to some extent. Meanwhile, the advantage of mechanical valves is that they can be used for life, but there are also some disadvantages, such as the need for lifelong use of anticoagulants and the obvious noise of the valve [13]. Considering the service life of valves and the relationships between valve type and religion, region, average lifespan of the population, different life values and life expectancy, mechanical prosthetic valves are the first choice for valve replacement in those patients aged under 60 years old in China [2]. The biological prosthetic valve is the first choice for valve replacement in elderly patients aged over 70 years because of life expectancy. For sexagenarian patients with aortic valve lesions, the choice of a mechanical valve means lifelong anticoagulation therapy, while the choice of a biological valve means the possibility of repeat surgery. Choosing the type of prosthetic valve is difficult for Chinese clinicians and patients. Advances in society have led people to care more about quality of life than length of life. In addition to considering the performance of the two prosthetic valves, the postoperative short-term and long-term complications, the survival of patients and the recurrence of the disease, quality of life has also become an important evaluation criterion in choosing the type of prosthetic valve [14, 15]. This study retrospectively analyzed the quality of life and postoperative anxiety of sexagenarian patients who underwent biological or mechanical aortic valve replacement in our center to provide some reference factors for valve selection for sexagenarian patients to be used in the future.

Through the SF-36 questionnaire survey, we found that the scores of the 7 fields in the biological valve group was significantly higher than those in the mechanical valve group at one-year follow-up, indicating that sexagenarian patients with aortic biological valves experienced better quality of life than those with mechanical valves. Although mechanical valves have longer durability than biological valves, they require anticoagulant therapy for life after surgery. Most patients who undergo prosthetic valve replacement understand the purpose and significance of the anticoagulation therapy as a preventive treatment measure that plays an important role in avoiding valve thrombosis [16–18]. However, the majority of patients are dissatisfied with needing to undergo postoperative anticoagulation therapy. This tedious process requires not only long-term use of anticoagulant drugs but also the detection of blood coagulation function regularly, which makes anticoagulation-related issues (hemorrhage and thrombosis) ominous in the patient’s daily life. Patients with mechanical valves need to take anticoagulant drugs every day after surgery and regularly come to the hospital to have blood tests. They are always at risk of anticoagulation-related complications, which greatly affect the quality of life of patients. Another factor that continues to affect the daily life of patients after mechanical aortic valve replacement is valve opening and closing noise [19, 20]. Many studies have also shown the effects of valve noise on patients, which can lead to anxiety, irritability and even sleep disorders. In addition, valve opening and closing sounds from mechanical valves were not only heard by the patients themselves but also by others [21]. This could also explain why not only patients but also their partners have sleep disorders [22].

Patients who have undergone biological aortic valve replacement may be more worried about disease progression due to possible biological valve decay and the need for secondary valve replacement than those who have undergone mechanical aortic valve replacement. In our cardiac anxiety questionnaire, we found the opposite results: patients with mechanical valve replacement showed greater anxiety than those with biological valve replacement. The fear, avoidance and attention scores in the mechanical valve group were significantly higher than those in the biological valve group. Such results were similar to the findings of Aicher, who also believe that aortic valve formation and biological valve replacement have less long-term impact on quality of life than mechanical aortic valve replacement [23]. To analyze the reasons for these results, we determined the main influencing factors: patient concern regarding continuous anticoagulant therapy and continuous valve opening and closing noise following mechanical aortic valve replacement. These factors had a greater impact on quality of life than the possibility of needing a second operation in the biological aortic valve group. In addition, China is a developing country, where the level of primary medical care is suboptimal, and the level of medical care in communities, especially in remote rural areas, is particularly lacking. Most patients with valvular diseases in China come from rural areas, and it is relatively difficult for them to seek medical treatment if there is an anticoagulation problem after the mechanical valve replacement; as such, patients are particularly worried that anticoagulation problems will occur, especially sexagenarian patients. These patients tend to lack medical knowledge, have no adequate medical counseling, and can have no children to help them, and with the memory decline that accompanies aging, the chance that these patients will forget to take their anticoagulants increases correspondingly. These findings may explain why patients in the biological aortic valve group scored higher than the mechanical valve group on the SF-36 and CAQ surveys in this study. Although age, complications, mortality, and specific prosthetic valvular defects may affect sexagenarian patient decisions when choosing between mechanical and biological valves, more consultation should be given to individuals, especially in terms of quality of life and psychological aspects.

There were some limitations in this study. First, this was a retrospective case study and lacked randomization. Second, this was a single-center study with a small sample size, different regions, different cultures, different beliefs might lead to different results [24]. Third, the follow-up time was short, and the quality of life of patients in other age groups and their long-term follow-up data were not studied; in particular, patients in the middle age group received biological valves for fear that the need for reoperation might increase and the quality of life might be affected with mechanical valves. And these might be why Sedrakyan and his team got a different conclusion from ours [24]. There was a statistically significant difference in the age composition of the two groups of patients in their study.

## Conclusion

The quality of life and anxiety scores of sexagenarian patients after aortic valve replacement were better in those wo received a biological valve than those who received a mechanical valve because patients who received a biological valve did not have to worry about the side effects of anticoagulants and the noise related to mechanical valves, even with the potential need for reoperation. Therefore, based on the results of the postoperative quality of life and CAQ surveys, it is more reasonable to choose a biological valve than a mechanical valve for sexagenarian patients undergoing aortic valve replacement.

## Data Availability

Data sharing not applicable to this article as no data sets were generated or analyzed during the current study.

## Abbreviations

SF-36: The Medical Outcomes Study Short Form 36
CAQ: Cardiac anxiety questionnaire
