# Correction to: Quality of life in sexagenarians after aortic biological vs mechanical valve replacement: a single-center study in China

**DOI:** 10.1186/s13019-020-01256-2

**Published:** 2020-08-03

**Authors:** Li-Wen Wang, Ning Xu, Shu-Ting Huang, Liang-Wan Chen, Hua Cao, Qiang Chen

**Affiliations:** 1 Department of Cardiac Surgery, Fujian Maternity and Child Health Hospital, Affiliated Hospital of Fujian Medical University, Fuzhou, China; 2 Department of Cardiovascular Surgery, Union Hospital, Fujian Medical University, Fuzhou, China

**Correction to: World Journal of Emergency Surgery 15, 88 (2020)**

**https://doi.org/10.1186/s13019-020-01143-w**

The original article [1] contains two errors in Table 1:

1. The age in Biological AVR group should instead be 65.6 +/− 4.3
2. The age in Mechanical AVR group should instead be 64.8 +/− 5.1
